# The effects of host ecology and phylogeny on gut microbiota (non)parallelism across birds and mammals

**DOI:** 10.1128/msphere.00442-23

**Published:** 2023-12-01

**Authors:** Andreas Härer, Diana J. Rennison

**Affiliations:** 1 Department of Ecology, Behavior & Evolution, School of Biological Sciences , University of California San Diego, La Jolla, California, USA; Kansas State University, Manhattan, Kansas, USA

**Keywords:** parallel evolution, gut microbiome, 16S rRNA gene sequencing, vertebrates, trophic ecology, multivariate vector analysis

## Abstract

The gut microbiota plays a crucial role in the biological function of their hosts since bacteria provide nutrient processing, protection against pathogens, and modulation of the immune system. Thus, as organisms adapt to different ecological niches, their gut microbial communities are thought to change in response to novel environmental conditions. Quantifying the direction and magnitude of gut microbiota changes associated with ecological niche offers valuable insights into the predictability of gut microbial community change accompanying shifts in host ecology. We used multivariate vector analysis to assess the (non)parallelism of gut microbiota community composition associated with host occupancy of a similar niche. We studied gut microbiota (non)parallelism across an extensive data set of 53 mammalian and 50 avian host species. Our results suggest that parallel shifts in host trophic ecology are associated with greater gut microbiota parallelism, whereas we could not find evidence for a clear effect of host phylogeny. Furthermore, parallelism was generally stronger when estimated from inferred metagenome function compared with gut microbiota taxonomic composition, suggesting that functional redundancy among bacterial lineages might obscure the signature of adaptive gut microbiota changes. Notably, a substantial proportion of the variation in gut microbiota (non)parallelism was not explained by host trophic ecology or phylogeny. Thus, we encourage future microbiota studies to use quantitative analyses along with additional ecological and host-associated data to obtain a comprehensive understanding of the factors shaping the direction and magnitude of gut microbiota changes associated with ecological divergence.

**IMPORTANCE:**

What are the roles of determinism and contingency in evolution? The paleontologist and evolutionary biologist Stephen J. Gould raised this question in his famous thought experiment of “replaying life’s tape.” Settings where independent lineages have repeatedly adapted to similar ecological niches (i.e., parallel evolution) are well suited to address this question. Here, we quantified whether repeated ecological shifts across 53 mammalian and 50 avian host species are associated with parallel gut microbiota changes. Our results indicate that parallel shifts in host diet are associated with greater gut microbiota parallelism (i.e., more deterministic). While further research will be necessary to obtain a comprehensive picture of the circumstances under which deterministic gut microbiota changes might be expected, our study can be instrumental in motivating the use of more quantitative methods in microbiota research. This, in turn, can help us better understand microbiota dynamics during adaptive evolution of their hosts.

## INTRODUCTION

Diverse microbial communities form associations with multicellular hosts, and these associations seem to be ubiquitous across the animal kingdom (1). Microbes appear to play a crucial role in their hosts’ ecology and evolution (2–5). Gut microbiota are thought to be particularly important as they impact their host’s physiology in multiple ways, including nutrient metabolism (6). Through these interactions, gut microbiota may be important for their hosts’ trophic niche (i.e., their dietary resources) adaptation (3, 7). Over the last few decades, gut microbiota from a broad range of organisms have been characterized, and this work has revealed the immense diversity of host-associated microbial communities (8–10). These rich data sets can be used to identify factors that structure gut microbiota variation across host lineages; for example, phylogeny, diet, and physiology were found to be critical in determining the composition of gut microbial communities (8, 11, 12). Leveraging this knowledge allows us to ask whether and under which circumstances we might be able to predict the direction and magnitude of gut microbiota changes (13). This question is best addressed in settings where independent host lineages have repeatedly adapted to similar ecological niches (i.e., the resources an organism requires and its position in the ecosystem), which is often accompanied by repeated changes in their trophic ecology, morphology, physiology, and potentially their gut microbiota (hereafter referred to as parallel evolution).

The roles of determinism and contingency during adaptive evolution have been debated for over 30 years, ever since S. J. Gould proposed his thought experiment of “replaying life’s tape.” Parallel evolution is commonly regarded as evidence for natural selection (14, 15), and cases of parallel evolution can be leveraged to investigate to what extent phenotypic changes associated with ecological shifts may be predicted (i.e., how deterministic they are). A particularly intriguing question is whether repeated ecological shifts across independent host lineages are associated with parallel changes of the gut microbiota. This question has been addressed in different lineages, with varying results (8, 16–18). One limitation of most previous studies is that they scored (non)parallelism as binary (parallel vs nonparallel) rather than quantifying the direction and magnitude of gut microbiota changes. Quantification of non(parallelism) can be achieved with multivariate vector analysis (19, 20), which has been previously used to estimate (non)parallelism for a broad range of phenotypic traits across multiple fish species (21–23). To date, only one study has applied this method for studying the gut microbiota (24), but the utility of multivariate vector analysis for studying patterns of gut microbiota variation has been suggested (13). We advocate for the use of quantitative methods, such as multivariate vector analysis, to determine the direction and magnitude of gut microbiota shifts across host lineages, which can help identify the ecological and evolutionary processes that shape variation in host-associated microbial communities. A broad application of this method would allow for tests of the conditions and factors with which gut microbiota parallelism might be expected. Furthermore, evidence of gut microbiota parallelism would suggest that changes of the gut microbiota are to some extent adaptive and, thus, predictable (13, 24).

To determine how different factors, in particular host ecology and phylogeny, contribute to the variation in gut microbial communities across their vertebrate hosts, large-scale data sets with phylogenetically diverse host species are imperative. Since diet has a strong effect on the composition of the gut microbiota (18, 25–28), one could expect to see stronger gut microbiota parallelism across host lineages with parallel divergence in trophic ecology. Phylogenetic distance (or divergence time, i.e., the time since two lineages shared a common ancestor) among host lineages has also been shown to affect the magnitude of gut microbiota divergence (8, 11, 12, 29), but how this affects gut microbiota (non)parallelism is currently unclear. We could predict that more recently diverged host lineages are more similar to each other in traits that affect the gut microbiota (genetic composition, gut morphology, and ecology) (11, 12, 30, 31), and hence, the direction and magnitude of gut microbiota divergence might be limited. This would potentially lead to stronger parallelism in more recently diverged hosts. However, it is also possible that gut microbiota changes among recently diverged host lineages might be more stochastic and that sufficient divergence in host traits is necessary to produce deterministic gut microbiota change, which would be reflected in stronger parallelism in more distantly related hosts.

**Fig 1:**
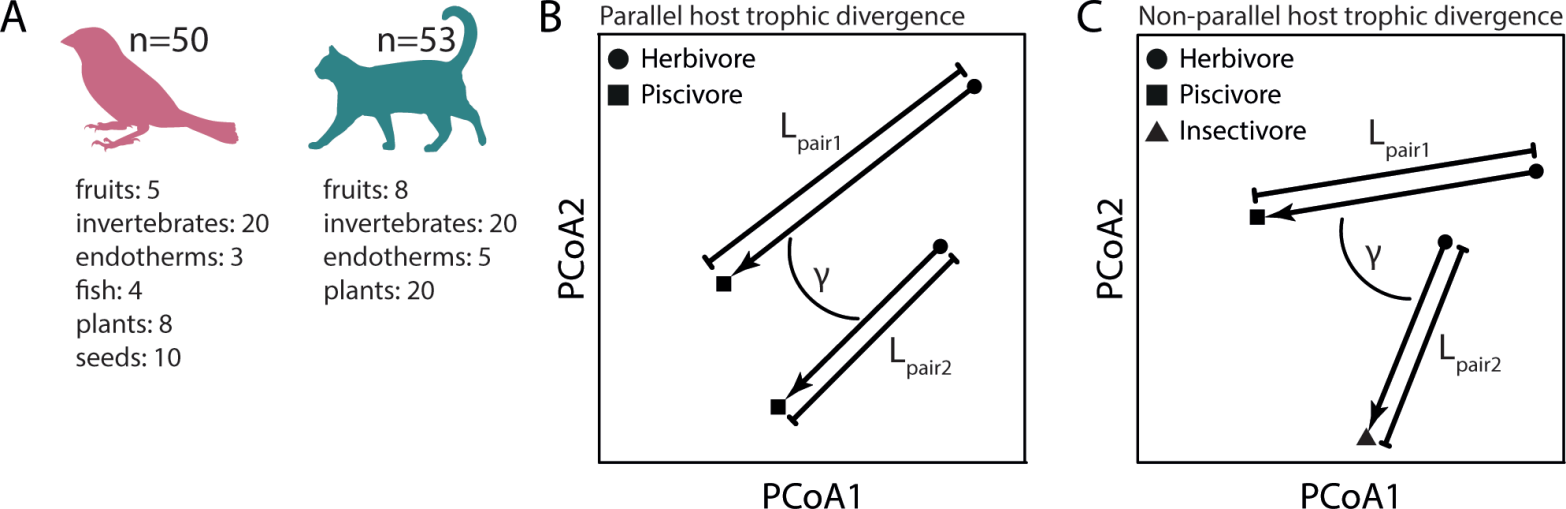
Our data set consisted of 50 avian and 53 mammalian species feeding on different diet types (**A**). Angles between vectors and vector lengths were calculated between species pairs to quantify direction and magnitude of gut microbiota changes associated with parallel (**B**) and nonparallel (**C**) changes in host trophic ecology. Avian and mammalian silhouettes were obtained from vecteezy.com.

To test these alternative predictions and identify factors contributing to variation in gut microbial communities across vertebrate hosts, we used multivariate vector analysis to quantify direction and magnitude of changes in gut microbiota taxonomic composition and inferred metagenome function across 53 mammalian and 50 avian host species (Fig. 1). This represents a subset of taxa and samples from a large-scale study by Song et al. (8). Specifically, we addressed the following questions: (i) Are parallel shifts in host trophic ecology associated with more parallel gut microbiota changes? (ii) Does divergence time and phylogenetic relationship between host lineages affect gut microbiota parallelism or the magnitude of gut microbiota change? (iii) Do direction and magnitude of change differ between gut microbiota composition and function? (iv) Do patterns of (non)parallelism differ between mammals and birds, as might be expected based on previous results (8)?

## MATERIALS AND METHODS

### Data acquisition and filtering

All analyses were based on published 16S rRNA gene sequencing data and metadata from Song et al. (8) that were obtained from github (https://github.com/tanaes/tetrapod_microbiome_analysis). Thus, we refer to Song et al. for details on data filtering and all upstream analyses. Samples originated from captive or wild animals (Tables S1 and S2). The amplicon sequence variant (ASV) table created by Song et al. was downloaded from github and then imported into the open-source bioinformatics pipeline QIIME2 (32), and distance matrices were produced for bacterial community composition based on the Bray-Curtis dissimilarity metric. To predict metagenome function, we obtained MetaCyc pathway abundances based on 16S rRNA gene sequence data using the PICRUSt2 plugin in QIIME2 with a maximum nearest-sequenced taxon index (NSTI) cutoff of 2 (33, 34).

The data were then filtered to remove duplicate samples of the same individual (due to different preservation methods) and samples that did not have an individual ID. We also removed diseased, injured, and unhealthy individuals, young and old individuals (only the following age categories were maintained: “adult,” “after hatch year,” and “unknown”), and samples with less than 1,000 deblurred sequences and species with less than three individuals. We further reduced the data sets to a maximum of 20 individuals per species, which were randomly selected. Since we were interested in the magnitude and direction of gut microbiota changes associated with changes in diet, we only kept species that are specialized in a specific type of diet. We defined a specialist as a species consuming 70% or more of one diet category; diet data were obtained from the metadata files associated with Song et al. (8) and included the following categories: fruits, invertebrates, meat-endotherms, meat-fish, plants, and seeds. These filtering steps left us with a total of 434 mammalian and 234 avian individuals belonging to 53 and 50 species, respectively (Tables S1 and S2). Mammalian and avian data sets were analyzed separately, and results were compared within and across these two vertebrate classes. Phylogenetic trees of all avian and mammalian host lineages and divergence times between them were obtained from TimeTree (35). All data files and R scripts have been deposited in the figshare database (https://doi.org/10.6084/m9.figshare.23841123.v1).

### Multivariate vector analysis

Since previous analyses have found that gut microbiota (non)parallelism estimates calculated with different metrics (Bray-Curtis dissimilarity, unweighted and weighted UniFrac) are highly correlated (13), we decided to only use one metric to quantify (non)parallelism for taxonomic composition (Bray-Curtis dissimilarity) and inferred metagenome function (MetaCyc pathway abundances based on Bray-Curtis dissimilarity). Using these filtered data sets, we performed principal coordinate analyses (PCoA) based on distance matrices. Only PCoA axes that explained greater than 0.1% of the variation were retained. We calculated mean PCoA scores across the retained axes for each species and connected these with multivariate vectors; see references (13) and (24) for more details on the methodology. These vectors provide a quantitative measure of direction (angles between vectors) and magnitude (vector length) in gut microbiota change between two pairs of host species (Fig. 1). In general, smaller angles indicate stronger parallelism, and angles below 90° are indicative of gut microbiota parallelism whereas angles above 90° are indicative of nonparallelism and 180° would represent anti-parallelism.

To test for differences in gut microbiota (non)parallelism among host groups (i.e., birds vs mammals), we used unpaired two-sample *t*-tests. We further tested for associations between the extent of gut microbiota (non)parallelism and mean divergence times between host lineages using linear models (lm function of the R stats package v4.2.1; 36) as well as generalized additive models (gam function of the R mgcv package v1.8–40; 37) to identify nonlinear variation. In all cases, the generalized additive models explained a higher proportion of the variance in angles; hence, only the results of these models are presented. We tested whether hosts’ parallel divergence in trophic ecology was associated with the extent of gut microbiota (non)parallelism. To address this question, we created two subsets of comparisons. In the “parallel host trophic divergence” category, the two members of each species pair had the same two types of diet (e.g., both pairs have one host species feeding on plants and one feeding on fish; Fig. 1). In contrast, in the “nonparallel host trophic divergence” category, the two members of each species pair had different diets (e.g., one pair comprises a host species that feeds on plants and the other one on fish whereas the second pair comprises a host species that feeds on plants and the other one on insects). To specifically focus on cases of trophic divergence across host lineages; for these analyses, we excluded species pairs where both host species had the same type of diet. All statistical analyses were done in R v4.2.1 (36).

For a set of subsequent analyses, we filtered the data sets to solely include “parallel host trophic divergence” comparisons for which the two different ecotypes in each species pair were more closely related to each other (Fig. 4A). This allowed us, to some extent, to investigate the effects of host ecology and phylogeny on gut microbiota (non)parallelism, by comparing angles between different comparisons within and across diet categories. Specifically, we calculated vectors either between the two different diet categories in a species pair (A_1_B_1_-A_2_B_2_), between the same diet categories across two species pairs (A_1_A_2_-B_1_B_2_), or between different diet categories across species pairs (A_1_B_2_-A_2_B_1_); see Fig. 4A for the depiction of these pairings. If angles were smallest for the A_1_B_1_-A_2_B_2_ comparisons, host ecology might be the major driver of gut microbiota parallelism. In contrast, if angles were smallest for the A_1_A_2_-B_1_B_2_ comparisons, host phylogeny might have a major effect on gut microbiota parallelism. For the third comparison (A_1_B_2_-A_2_B_1_), angles were expected to be the smallest if a combined effect of host ecology and phylogeny produced the strongest gut microbiota parallelism. Due to nonnormal distribution of the data based on Shapiro-Wilk tests (38), we used nonparametric Kruskal-Wallis tests, followed by pairwise Wilcoxon rank-sum tests to test for differences in angles among types of comparisons.

Finally, we quantified the magnitude of gut microbiota divergence by calculating vector lengths for each species pair (Fig. 1). To test for differences in vector length between host groups and diet categories, we used the nonparametric Wilcoxon rank-sum test (39) since data were not normally distributed based on Shapiro-Wilk tests (38). We further tested for differences in vector length associated with trophic divergence of the host species. For this, species pairs were either scored as “same diet” (i.e., the two members feed on the same diet) or “different diet” (i.e., the two members feed on different diets).

## RESULTS

### Direction of gut microbiota divergence: angles between vectors

#### Taxonomic composition

**Fig 2:**
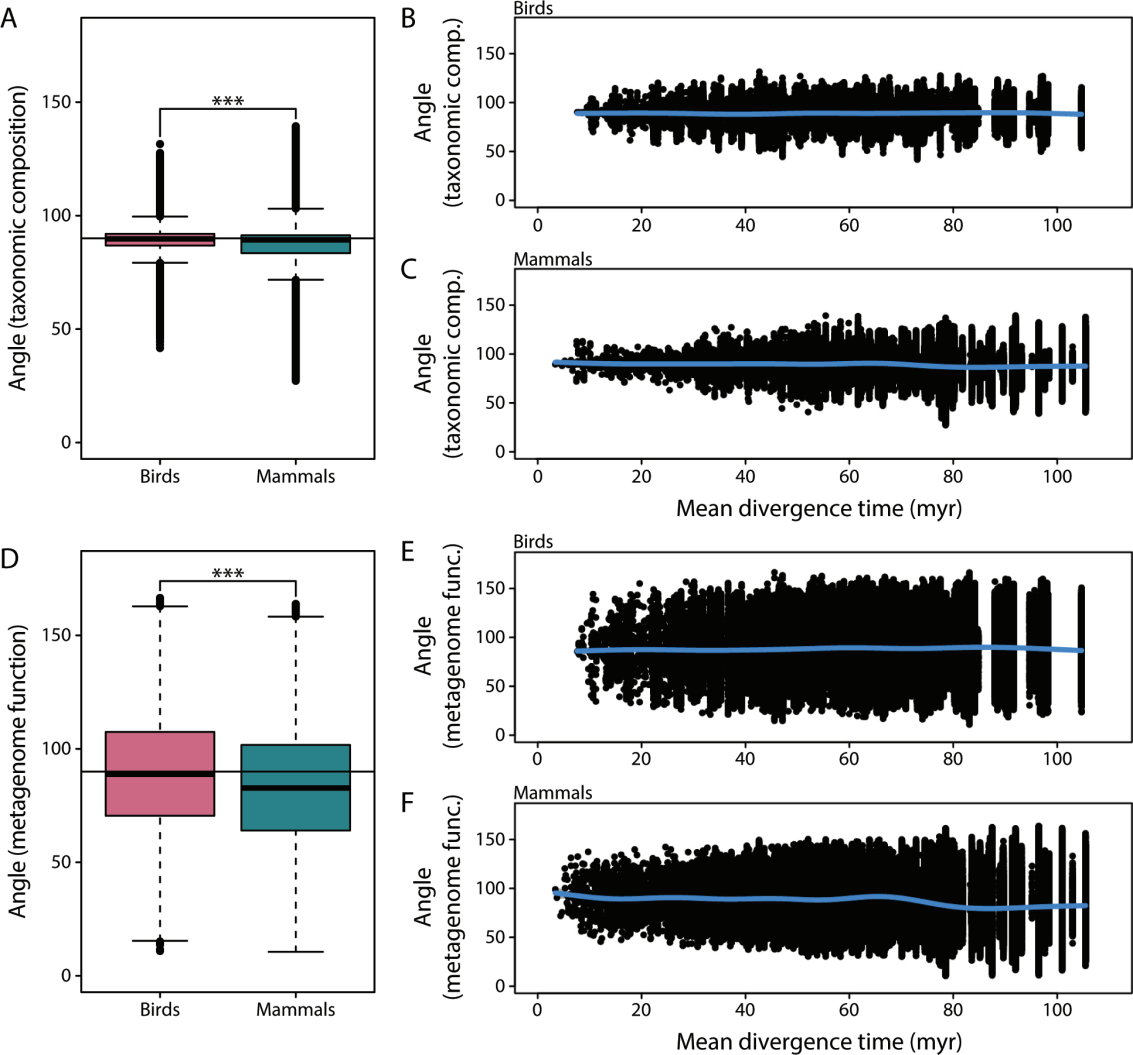
Across all comparisons, parallelism was stronger (i.e., smaller angles between vectors) in mammals compared with birds for the taxonomic composition of the gut microbiota (**A**) and inferred metagenome function (**D**). Angles were only weakly associated with host divergence time in birds (B, E) and mammals (C, F); generalized additive models are illustrated to identify nonlinear variation. ***P < 0.001.

For the taxonomic composition of the gut microbiota, angles showed a normal distribution centered at 90° in birds and mammals which approximately follows the expected distribution of random angles in multidimensional space (40). Angles were smaller in mammals (mean: 87.64°) than in birds (mean: 89.25°; two-sample *t*-test: *P* < 0.001, *t* = 120) ( Fig. 2A). At the same time, mean host divergence times (obtained from TimeTree) for species pair comparisons were longer for mammals (mean: 82.06 million years (myr)) compared with birds (mean: 77.82; two-sample *t*-test: *P* < 0.001, *t* = −172.8) (Fig. S1). Angles differed with host divergence time in birds (generalized additive model: *P* < 0.001, *R*^2^ = 0.003) and mammals (generalized additive model: *P* < 0.001, *R*^2^ = 0.017). Yet, these models only explained a very small proportion of the variance in angles and no clear direction of these associations (i.e., positive or negative) could be determined (Fig. 2B and C). Notably, small and large angles appeared to be largely missing for more recently diverged species pairs as illustrated by the lower variation and tighter clustering of angles (e.g., compare distribution of angles at divergence times below 20 myr to those between 40 and 100 myr in (Fig. 2B and C). Together, these results indicate slight differences in the distribution of angles between birds and mammals, but it remains unclear whether there is a positive or negative association between host divergence time and parallelism estimates.

**Fig 3:**
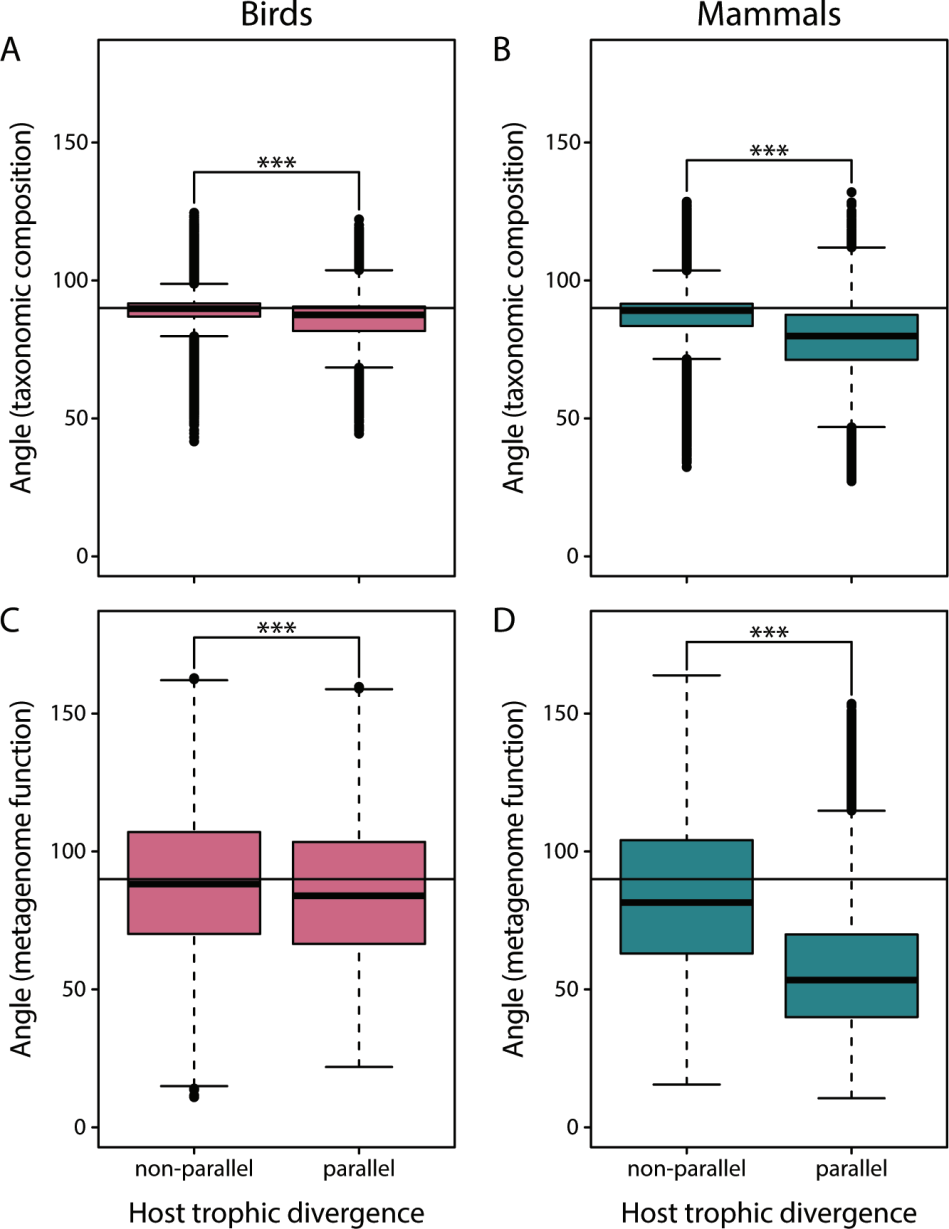
Parallelism in the gut microbiota’s taxonomic composition (A, B) and inferred metagenome function (C, D) is stronger (i.e., angles are smaller) when trophic divergence of host lineages is parallel in both birds (A, C) and mammals (B, D). Parallel host trophic divergence is defined as settings in which members of each species pair feed on the same two types of diet (Fig. 1). ***P < 0.001.

We created two subsets of comparisons, one in which the two members of each species pair are in the same two diet categories (parallel host trophic divergence) and one in which the two members of each species pair are in two different diet categories (nonparallel host trophic divergence) (Fig. 1; see Materials and Methods for more details). Angles were smaller for comparisons in the “parallel host trophic divergence” category compared with the “nonparallel host trophic divergence” category, both in birds (mean: 85.85° vs 89.05°; two-sample *t*-test: *P* < 0.001, *t* = 75.8) (Fig. 3A) and in mammals (mean: 78.12° vs 88.04°; two-sample *t*-test: *P* < 0.001, *t* = 245.8) (Fig. 3B). Divergence times also differed between the two categories; divergence times were shorter for the “parallel host trophic divergence” category in birds (two-sample *t*-test: *P* < 0.001, *t* = 42.2) but shorter for the “nonparallel host trophic divergence” category in mammals (two-sample *t*-test: *P* < 0.001, *t* = −86.8) (Fig. S1). For birds, we found evidence for an effect of host divergence time on parallelism within the “nonparallel host trophic divergence” category (generalized additive model: *P* < 0.001, *R*^2^ = 0.006), but the direction of this association was unclear (Fig. S2A). For the “parallel host trophic divergence” category, angles appeared to increase with host divergence time (generalized additive model: *P* < 0.001, *R*^2^ = 0.082) and host divergence time explained a larger proportion of the variance in angles compared with the “nonparallel host trophic divergence” category (Fig. S2B). For mammals, angles were rather constant across host divergence times in the “nonparallel host trophic divergence” category (generalized additive model: *P* < 0.001, *R*^2^ = 0.025) (Fig. S2C). In the “parallel host trophic divergence” category, angles appeared to decrease for small host divergence times and then increased for large divergence times (generalized additive model: *P* < 0.001, *R*^2^ = 0.086), and similar to birds, host divergence time explained a larger proportion of the variance for this category (Fig. S2D).

**Fig 4:**
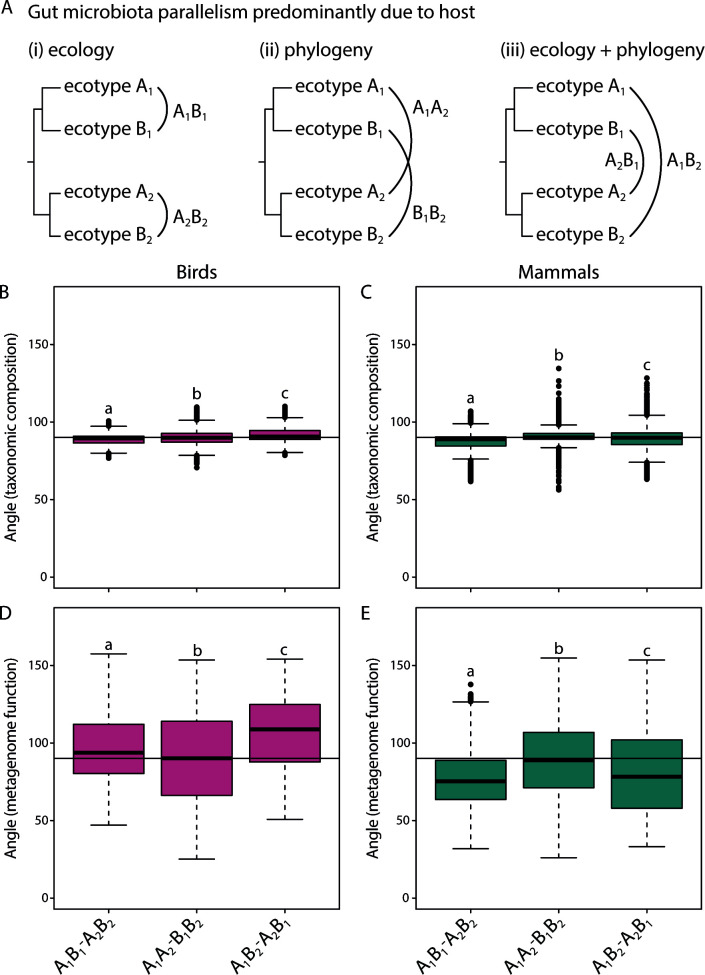
Only considering “parallel host trophic divergence” comparisons for which the two different ecotypes in each species pair were more closely related to each other allowed us to determine whether host ecology or phylogeny has a stronger effect on gut microbiota parallelism (**A**). Angles were generally smallest for the A_1_B_1_-A_2_B_2_ comparisons (except for inferred metagenome function in birds), suggesting that host ecology has the strongest effect on parallelism in gut microbiota changes (**B–E**). Different letters indicate significant differences between types of comparisons (*P* < 0.05).

Next, we only considered “parallel host trophic divergence” species pair comparisons for which the two members of each species pairs were most closely related to each other (Fig. 4A). For this subset, we calculated angles for three different comparison types: (i) between the closely related species pairs that differ in their trophic ecology (A_1_B_1_-A_2_B_2_) and between more distantly related species pairs with (ii) similar trophic ecology (A_1_A_2_-B_1_B_2_) or (iii) different trophic ecology (A_1_B_2_-A_1_B_2_; Fig. 4A). Creating these subsets allows tests of the effects of host ecology and phylogeny on gut microbiota (non)parallelism. Angles differed significantly across comparison types in birds (Kruskal-Wallis test: χ^2^ = 235.2, *P* < 0.001) (Fig. 4B) and mammals (Kruskal-Wallis test: χ^2^ = 501.9, *P* < 0.001) (Fig. 4C) for gut microbiota taxonomic composition. Post hoc Wilcoxon rank-sum tests showed that angles differed between all types of pairwise comparisons in birds and mammals, and on average, the A_1_B_1_-A_2_B_2_ comparisons had the smallest angles (mean_birds_: 88.91°, mean_mammals_: 87.35°) compared with the A_1_A_2_-B_1_B_2_ (mean_birds_: 89.93°, mean_mammals_: 90.90°) and A_1_B_2_-A_1_B_2_ comparisons (mean_birds_: 91.63°, mean_mammals_: 89.33°) (Fig. 4B and C).

#### Inferred metagenome function

Similar to the taxonomic composition of the gut microbiota, angles based on inferred metagenome function were smaller in mammals (mean: 83.09°) than in birds (mean: 89.09°; two-sample *t*-test: *P* < 0.001, *t* = 146.2) (Fig. 2D). Angles also differed with host divergence time, but again, no clear trend was observable in birds (generalized additive model: *P* < 0.001, *R*^2^ = 0.001) (Fig. 2E) or mammals (generalized additive model: *P* < 0.001, *R*^2^ = 0.024) (Fig. 2F), and the proportion of variance explained by these models was comparable to the result for gut microbiota taxonomic composition. Notably, angles were significantly smaller for inferred metagenome function than for taxonomic composition in birds (two-sample *t*-test: *P* < 0.001, *t* = 5.1) and mammals (two-sample *t*-test: *P* < 0.001, *t* = 151.9). Yet, it should be noted that the mean angles differed only slightly in birds (89.09° vs 89.25°) but more so in mammals (83.09° vs 87.64°; Fig. S3). The distribution of angles was also much broader for inferred metagenome function compared taxonomic composition (Fig. S3).

Between the parallel and nonparallel host trophic divergence categories, angles were smaller for the “parallel host trophic divergence” category in birds (mean: 85.37° vs 88.62°; two-sample *t*-test: *P* < 0.001, *t* = 24.8) (Fig. 3C) and much more so in mammals (mean: 56.86° vs 83.94°; two-sample *t*-test: *P* < 0.001, *t* = 317) (Fig. 3D) based on inferred metagenome function. For birds, angles varied with host divergence time in both the “nonparallel host trophic divergence” category (generalized additive model: *P* < 0.001, *R*^2^ = 0.001) (Fig. S4A) and the “parallel host trophic divergence” category (generalized additive model: *P* < 0.001, *R*^2^ = 0.018) (Fig. S4B) but the proportion of variance explained was much larger for the “parallel host trophic divergence” category. For mammals, there was again no clear trend observed for the “nonparallel host trophic divergence” category (generalized additive model: *P* < 0.001, *R*^2^ = 0.036) (Fig. S4C), but angles decreased with mean host divergence times in the “parallel host trophic divergence” category (generalized additive model: *P* < 0.001, *R*^2^ = 0.082) (Fig. S4D).

Similar to the taxonomic composition, we limited our analysis to “parallel host trophic divergence” species pair comparisons for which the two different ecotypes in each species pairs were most closely related to each other to disentangle the effects of host trophic ecology and phylogeny on parallelism estimates. We found that angles also differed across types of comparisons for inferred metagenome function in birds (Kruskal-Wallis test: χ^2^ = 285.7, *P* < 0.001) (Fig. 4D) and mammals (Kruskal-Wallis test: χ^2^ = 390.9, *P* < 0.001) (Fig. 4E). In birds, angles of the A_1_A_2_-B_1_B_2_ comparisons were the smallest (mean_birds_: 90.23°, mean_mammals_: 89.04°); in mammals, the A_1_B_1_-A_2_B_2_ comparisons had the smallest angles (mean_birds_: 96.60°, mean_mammals_: 76.44°) whereas the A_1_B_2_-A_1_B_2_ comparisons were the largest in birds and intermediate in mammals (mean_birds_: 105.93°, mean_mammals_: 80.56°) (Fig. 4D and E).

### Magnitude of gut microbiota divergence: vector lengths

#### Taxonomic composition

**Fig 5:**
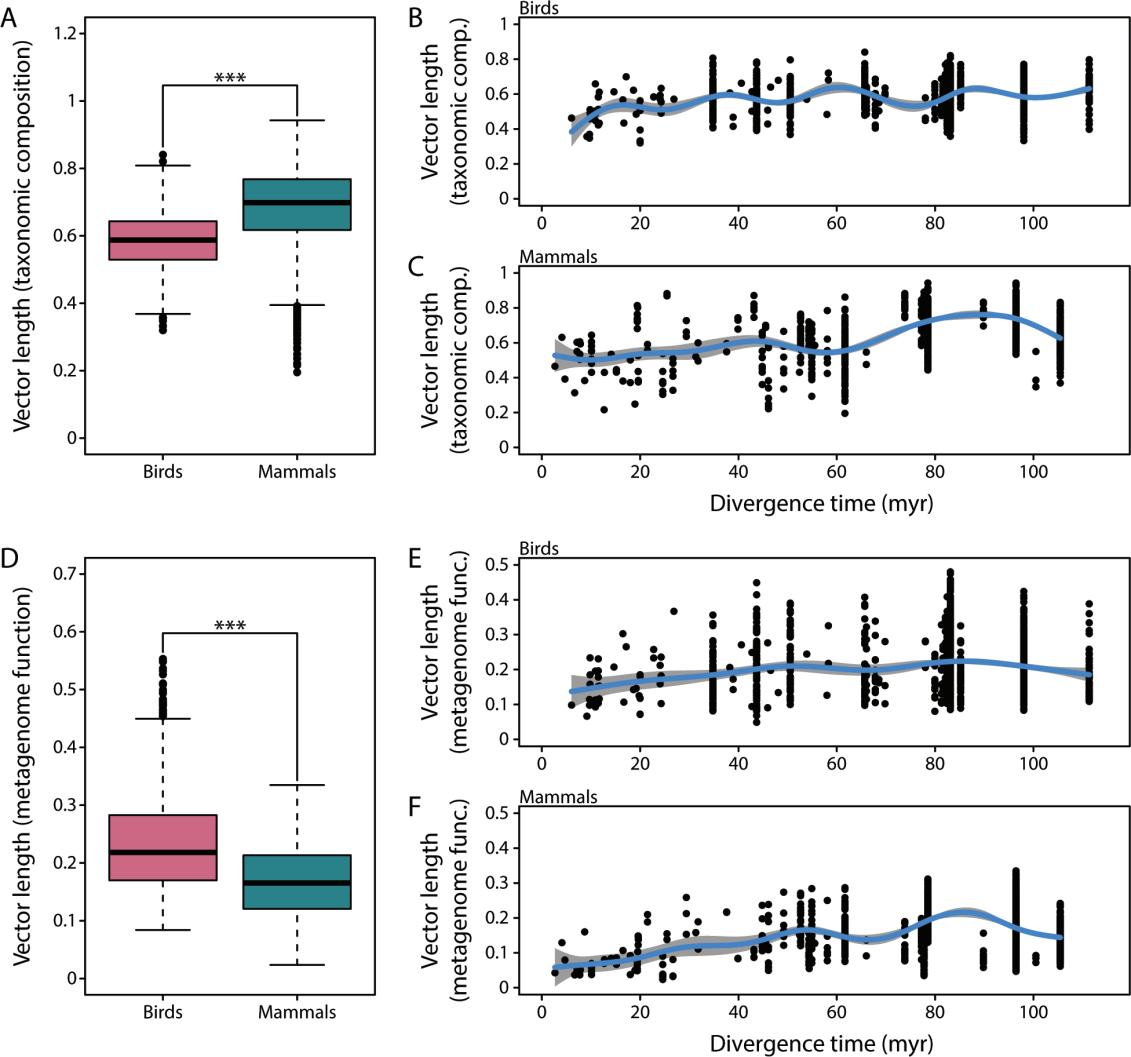
The extent of gut microbiota divergence (i.e., vector lengths) was smaller in birds for the taxonomic composition of the gut microbiota (**A**) but larger for inferred metagenome function (**D**). Vector length appeared to be positively associated with host divergence time in birds (B, E) and mammals (C, F); generalized additive models are illustrated to identify nonlinear variation. ***P < 0.001.

**Fig 6:**
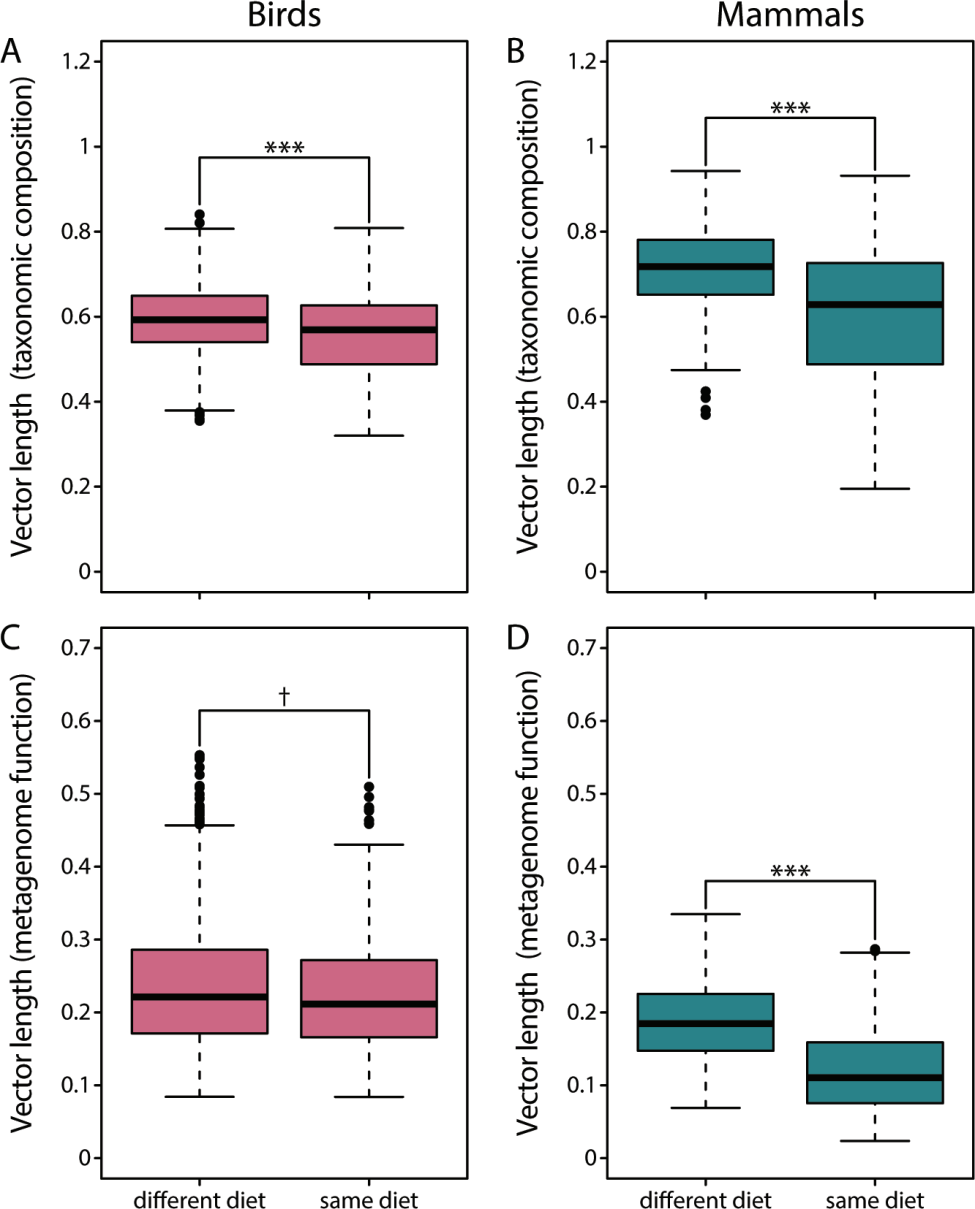
Vectors were longer when the two members of a species pair fed on the different types of diet, indicating higher extent of gut microbiota divergence. This was a consistent pattern found across birds (A, C) and mammals (B, D), as well as for the taxonomic composition of the gut microbiota (A, B) and inferred metagenome function (C, D). ^†^*P* < 0.1 and ***P < 0.001.

For the taxonomic composition of the gut microbiota, vectors were shorter in birds than in mammals (Wilcoxon rank-sum test: *P* < 0.001) (Fig. 5A), and this pattern was not driven by host divergence time of species pairs since these did not differ between birds and mammals (Wilcoxon rank-sum test: *P* = 0.484). The extent of gut microbiota divergence differed with host divergence time in birds (generalized additive model: *P* < 0.001, *R*^2^ = 0.066) (Fig. 5B) and mammals (generalized additive model: *P* < 0.001, *R*^2^ = 0.346) (Fig. 5C); vectors were generally shorter in more recently diverged species pairs. Vectors were also longer when the two members of a species pair fed on different diets in birds (Wilcoxon rank-sum test: *P* < 0.001, *t* = 5.6) (Fig. 6A) and mammals (Wilcoxon rank-sum test: *P* < 0.001; Fig. 6B). However, host divergence times were also longer for the “different diet” category in birds (Wilcoxon rank-sum test: *P* < 0.001) and mammals (Wilcoxon rank-sum test: *P* < 0.001), suggesting that the differences between “same diet” and “different diet” categories might, to some extent, be due to differences in host divergence time.

#### Inferred metagenome function

For inferred metagenome function, vectors were longer in birds than in mammals (Wilcoxon rank-sum test: *P* < 0.001) (Fig. 5D), contrary to the pattern observed for gut microbiota taxonomic composition (Fig. 5A). Vector length also differed with host divergence time in birds (generalized additive model: *P* < 0.001, *R*^2^ = 0.047) (Fig. 5E) and mammals (generalized additive model: *P* < 0.001, *R*^2^ = 0.169) (Fig. 5F), and vectors again appeared to be shorter in more recently diverged species pairs. Vectors were significantly longer for the “different diet” category in mammals (Wilcoxon rank-sum test: *P* < 0.001) (Fig. 6D), but there was only suggestive evidence for a difference in vector lengths in birds (Wilcoxon rank-sum test: *P* = 0.097) (Fig. 6C). In general, vector lengths were much larger for taxonomic composition of the gut microbiota than for inferred metagenome function in birds (Wilcoxon rank-sum test: *P* < 0.001) and mammals (Wilcoxon rank-sum test: *P* < 0.001).

## DISCUSSION

Using multivariate vector analysis (13, 19, 41), we quantified variation in the extent of gut microbiota (non)parallelism (angles between vectors) and divergence (vector lengths) for taxonomic and inferred functional gut microbiota changes across an extensive data set of 53 mammalian and 50 avian host species. Overall, we found that the distribution of angles differed between the two taxonomic groups and angles were generally smaller in mammals than in birds (Fig. 2). Since this pattern was found for species pair comparisons across all 53 mammalian and 50 avian host species and was independent of differences in host ecology (i.e., not taking into account whether host trophic divergence was parallel or nonparallel), it suggests that gut microbiota assembly is generally structured differently across these taxa. This is in line with results from Song et al. (8), which found stronger effects of host diet and phylogeny on gut microbiota composition in mammals than in birds (8). Furthermore, Song et al. showed that microbial communities associated with mammalian hosts were structured according to host taxonomy whereas little support for host specificity was detected in birds. Thus, changes in the composition of gut microbial communities across host species appear more deterministic in mammals, which might have contributed to the observed differences in the distribution of angles between mammals and birds. We currently cannot make inferences about the biological reasons for this pattern. This result raises the question of what the appropriate null hypothesis is when testing for microbiota (non)parallelism. Previous studies have shown that random angles in multidimensional space are generally expected to follow a normal distribution centered around 90° (40), and we observed a similar distribution in our study. However, our results suggest that differences among groups of host organisms should perhaps be considered when formulating null hypotheses for microbiota (non)parallelism studies.

Gut microbiota parallelism and divergence were affected by the similarity of host trophic ecology (as well as by divergence time and phylogenetic relationship of the hosts), which is likely representative of the similarity of components of the selective landscape, suggesting that similar gut microbiota changes might be adaptive. We detected smaller angles for comparisons where each vector pair consists of host species with the same two types of diet (i.e., the “parallel host trophic divergence” category; Fig. 1) indicating that host trophic ecology can be leveraged to predict changes in gut microbiota composition (Fig. 3). At the same time, there was less divergence (i.e., shorter vector lengths) when vectors consisted of two species with the same diet compared with vectors consisting of two species with different diets (Fig. 6). These results are not unexpected since diet is known to strongly affect the gut microbiota (26–28), but only two studies, to the best of our knowledge, have tested for gut microbiota parallelism using multivariate vector analysis to date (13, 24).

Angles were smaller for inferred metagenome function compared with taxonomic composition in the “parallel host trophic divergence” category of mammalian hosts (Fig. 3D). Similar results have been obtained in previous gut microbiota studies (13, 24). One potential explanation for this pattern is that the assembly of microbial communities can be strongly affected by historical contingencies (42). For example, the assembly of microbial communities can strongly depend on the order and timing of the arrival of different bacterial lineages and the probability of the establishment of bacterial lineages depends on which other bacteria are already present in this community. This phenomenon is referred to as “priority effect” and has been described to be a crucial determinant of microbial community composition (43). However, similar metabolic functions can be provided by distinct bacterial lineages due to functional redundancy (44). This, in turn, could lead to more deterministic changes and, hence, stronger parallelism based on inferred metagenome function associated with parallel changes in host trophic ecology.

The signal of parallelism accompanying trophic parallelism was highly variable; in the “parallel host trophic divergence” category, angles spanned from 44.5 to 122.2° in birds and 27.2–132° in mammals for gut microbiota taxonomic composition; the range of angles was even larger for inferred metagenome function (Fig. 3). This pattern indicates that, despite the overall stronger parallelism, a large proportion of the variation in angles is not explained by broad categorization of host trophic ecology. This trophic categorization used here is based on information regarding the major dietary component of a host species (e.g., plants, seeds, invertebrates, and fish) obtained from Song et al. (8). However, this coarse classification represents an oversimplification of diet similarity, as a diet category such as “plants” comprises an immense diversity of organisms and nutrients. Hence, we expect that more detailed information on host diet (ideally including nutritional profiles), described on the individual host level, would lead to stronger and more consistent estimates of gut microbiota parallelism. This prediction remains to be tested and future studies could use multivariate vector analysis to explicitly test to what extent some multidimensional description of diet similarity predicts gut microbiota similarity.

There is a range of factors that might contribute to the large variation in angles we observed in the “parallel host trophic divergence” category. For example, the broad diet categorization does not account for temporal and spatial variation in diet, which can strongly affect host-associated microbial communities (25, 45, 46). Besides, there is a plethora of technical (source material), host-associated (host morphology, physiology, and genetics) or environmental factors (abiotic conditions, geographic distribution) that can affect microbial community composition (5, 12, 47, 48) and the resulting (non)parallelism estimates. An important next step would be to more rigorously test how parallel changes in specific host-associated or environmental factors, such as changes in salinity or temperature that have been shown to affect the composition of host-associated microbial communities (49, 50), might affect microbiota (non)parallelism. While the main goal of our study was to identify general patterns associated with host trophic ecology and phylogeny and highlight the utility of multivariate vector analysis in microbiota research, it will be useful to determine for a range of host lineages how exactly a broad range of factors that contribute to the selective landscape affects gut microbiota (non)parallelism.

Besides trophic ecology, another crucial factor influencing the extent of gut microbiota (non)parallelism is the divergence time between host species. It is known that differentiation of microbial communities can be strongly associated with host phylogeny (9, 11). So, we aimed to disentangle the effects of host trophic ecology and phylogeny by creating subsets of vectors within the “parallel host trophic divergence” category. We found that host trophic ecology appeared to be the stronger driver of parallelism compared with host phylogeny (Fig. 4), lending further support to the notion that diet is a key predictor of gut microbiota composition. Our results did not show conclusive evidence for an association between host divergence time and the magnitude of parallelism. Yet, variation in angles appeared to be smaller for species pair comparisons with more recent divergence times, and small and large angles were largely missing from these comparisons (Fig. 2). This pattern was consistent across birds and mammals and also for both taxonomic composition and inferred metagenome function (Fig. 2). Furthermore, the extent of gut microbiota divergence (vector lengths) was positively associated with host divergence time, independent of host trophic ecology (Fig. 5). Taken together, these results suggest that the direction of gut microbiota change might be more restricted in recently diverged species and that observed changes are rather stochastic. This could possibly be due to less pronounced divergence in host ecology, morphology, and genetics (30, 51). Thus, we hypothesize that a minimum level of host divergence is necessary to observe gut microbiota parallelism in association with parallel changes in host trophic ecology.

Our study is the first to use multivariate vector analysis to test the effects of trophic ecology and divergence time of host lineages on the direction and magnitude of gut microbiota changes. This method can be used to determine the predictability of gut microbiota shifts across host lineages, thereby offering the opportunity to improve our understanding of the eco-evolutionary processes shaping the assembly of microbial communities. In accordance with previous studies, we found that the adaptation of host lineages to similar trophic niches is to some extent associated with predictable (i.e., more parallel) changes of their gut microbiota. Thus, multivariate vector analysis appears to produce biologically meaningful results and therefore likely represents a useful tool to study gut microbiota (non)parallelism. Yet, a substantial fraction of variation in gut microbiota (non)parallelism remained unexplained by host trophic ecology or phylogeny. Additional studies will be necessary to provide a more detailed picture of how the combined effects of a range of host-associated and environmental factors affect the distribution of microbiota diversity across host lineages, in particular finer scale diet data. Hence, we advocate using quantitative analyses in microbiota research which has the potential to substantially improve our understanding of which factors might allow predicting changes in gut microbiota composition. Ultimately, unraveling gut microbiota parallelism provides a comprehensive perspective on the complex interplay between hosts and their microbial partners during ecological divergence.

## SUPPLEMENTAL MATERIAL

Supplemental Material msphere.00442-23-s0001.docx Figures S1-S4 and captions for Tables S1 and S2.

Table S1 msphere.00442-23-s0002.csv Summary of all avian species analyzed in our study.

Table S2 msphere.00442-23-s0003.csv Summary of all mammalian species analyzed in our study.

ASM does not own the copyrights to Supplemental Material that may be linked to, or accessed through, an article. The authors have granted ASM a non-exclusive, world-wide license to publish the Supplemental Material files. Please contact the corresponding author directly for reuse.

## References

[1] McFall-Ngai M, Hadfield MG, Bosch TCG, Carey HV, Domazet-Lošo T, Douglas AE, Dubilier N, Eberl G, Fukami T, Gilbert SF, et al.. 2013. Animals in a bacterial world, a new imperative for the life sciences. Proc Natl Acad Sci USA 110:3229–3236. doi:10.1073/pnas.1218525110 23391737 PMC3587249

[2] Rudman SM, Greenblum S, Hughes RC, Rajpurohit S, Kiratli O, Lowder DB, Lemmon SG, Petrov DA, Chaston JM, Schmidt P. 2019. Microbiome composition shapes rapid genomic adaptation of Drosophila melanogaster. Proc Natl Acad Sci USA 116:20025–20032. doi:10.1073/pnas.1907787116 31527278 PMC6778213

[3] Zepeda Mendoza ML, Xiong Z, Escalera-Zamudio M, Runge AK, Thézé J, Streicker D, Frank HK, Loza-Rubio E, Liu S, Ryder OA, et al.. 2018. Hologenomic adaptations underlying the evolution of sanguivory in the common vampire bat. Nat Ecol Evol 2:659–668. doi:10.1038/s41559-018-0476-8 29459707 PMC5868727

[4] Sharpton TJ. 2018. Role of the gut microbiome in vertebrate evolution. mSystems 3:e00174-17. doi:10.1128/mSystems.00174-17 29629413 PMC5881020

[5] Härer A, Rennison DJ. 2023. The biogeography of host-associated bacterial microbiomes: revisiting classic biodiversity patterns. Global Ecol Biogeogr 32:931–944. doi:10.1111/geb.13675

[6] Turnbaugh PJ, Ley RE, Mahowald MA, Magrini V, Mardis ER, Gordon JI. 2006. An obesity-associated gut microbiome with increased capacity for energy harvest. Nature 444:1027–1031. doi:10.1038/nature05414 17183312

[7] Alberdi A, Aizpurua O, Bohmann K, Zepeda-Mendoza ML, Gilbert MTP. 2016. Do vertebrate gut metagenomes confer rapid ecological adaptation. Trends Ecol Evol 31:689–699. doi:10.1016/j.tree.2016.06.008 27453351

[8] Song SJ, Sanders JG, Delsuc F, Metcalf J, Amato K, Taylor MW, Mazel F, Lutz HL, Winker K, Graves GR, et al.. 2020. Comparative analyses of vertebrate gut microbiomes reveal convergence between birds and bats. mBio 11:e02901-19. doi:10.1128/mBio.02901-19 PMC6946802 31911491

[9] Youngblut ND, Reischer GH, Walters W, Schuster N, Walzer C, Stalder G, Ley RE, Farnleitner AH. 2019. Host diet and evolutionary history explain different aspects of gut microbiome diversity among vertebrate clades. Nat Commun 10:2200. doi:10.1038/s41467-019-10191-3 31097702 PMC6522487

[10] Sullam KE, Essinger SD, Lozupone CA, O’Connor MP, Rosen GL, Knight R, Kilham SS, Russell JA. 2012. Environmental and ecological factors that shape the gut bacterial communities of fish: a meta-analysis. Mol Ecol 21:3363–3378. doi:10.1111/j.1365-294X.2012.05552.x 22486918 PMC3882143

[11] Brooks AW, Kohl KD, Brucker RM, van Opstal EJ, Bordenstein SR. 2016. Phylosymbiosis: relationships and functional effects of microbial communities across host evolutionary history. PLoS Biol 14:e2000225. doi:10.1371/journal.pbio.2000225 27861590 PMC5115861

[12] Amato KR, G Sanders J, Song SJ, Nute M, Metcalf JL, Thompson LR, Morton JT, Amir A, J McKenzie V, Humphrey G, et al.. 2019. Evolutionary trends in host physiology outweigh dietary niche in structuring primate gut microbiomes. ISME J 13:576–587. doi:10.1038/s41396-018-0175-0 29995839 PMC6461848

[13] Härer A, Rennison DJ. 2022. Quantifying (non)parallelism of gut microbial community change using multivariate vector analysis. Ecol Evol 12:e9674. doi:10.1002/ece3.9674 36590339 PMC9797641

[14] Rosenblum EB, Parent CE, Diepeveen ET, Noss C, Bi K. 2017. Convergent phenotypic evolution despite contrasting demographic histories in the fauna of white sands. Am Nat 190:S44–S56. doi:10.1086/692138 28731825

[15] Losos JB, Jackman TR, Larson A, Queiroz K, Rodriguez-Schettino L. 1998. Contingency and determinism in replicated adaptive radiations of island lizards. Science 279:2115–2118. doi:10.1126/science.279.5359.2115 9516114

[16] Sevellec M, Derome N, Bernatchez L. 2018. Holobionts and ecological speciation: the intestinal microbiota of lake whitefish species pairs. Microbiome 6:47. doi:10.1186/s40168-018-0427-2 29540239 PMC5853090

[17] Baldo L, Pretus JL, Riera JL, Musilova Z, Bitja Nyom AR, Salzburger W. 2017. Convergence of gut microbiotas in the adaptive radiations of African cichlid fishes. ISME J 11:1975–1987. doi:10.1038/ismej.2017.62 28509910 PMC5560477

[18] Härer A, Torres-Dowdall J, Rometsch SJ, Yohannes E, Machado-Schiaffino G, Meyer A. 2020. Parallel and non-parallel changes of the gut microbiota during trophic diversification in repeated young adaptive radiations of sympatric cichlid fish. Microbiome 8:149. doi:10.1186/s40168-020-00897-8 33121541 PMC7597055

[19] Collyer ML, Adams DC. 2007. Analysis of two-state multivariate phenotypic change in ecological studies. Ecology 88:683–692. doi:10.1890/06-0727 17503596

[20] Adams DC, Collyer ML. 2009. A general framework for the analysis of phenotypic trajectories in evolutionary studies. Evolution 63:1143–1154. doi:10.1111/j.1558-5646.2009.00649.x 19210539

[21] Oke KB, Rolshausen G, LeBlond C, Hendry AP. 2017. How parallel is parallel evolution? a comparative analysis in fishes. Am Nat 190:1–16. doi:10.1086/691989 28617637

[22] Ingley SJ, Billman EJ, Hancock C, Johnson JB. 2014. Repeated geographic divergence in behavior: a case study employing phenotypic trajectory analyses. Behav Ecol Sociobiol 68:1577–1587. doi:10.1007/s00265-014-1767-y

[23] Stuart YE, Veen T, Weber JN, Hanson D, Ravinet M, Lohman BK, Thompson CJ, Tasneem T, Doggett A, Izen R, Ahmed N, Barrett RDH, Hendry AP, Peichel CL, Bolnick DI. 2017. Contrasting effects of environment and genetics generate a continuum of parallel evolution. Nat Ecol Evol 1:158. doi:10.1038/s41559-017-0158 28812631

[24] Rennison DJ, Rudman SM, Schluter D. 2019. Parallel changes in gut microbiome composition and function during colonization, local adaptation and ecological speciation. Proc Biol Sci 286:20191911. doi:10.1098/rspb.2019.1911 31795865 PMC6939261

[25] Smits SA, Leach J, Sonnenburg ED, Gonzalez CG, Lichtman JS, Reid G, Knight R, Manjurano A, Changalucha J, Elias JE, Dominguez-Bello MG, Sonnenburg JL. 2017. Seasonal cycling in the gut microbiome of the Hadza hunter-gatherers of Tanzania. Science 357:802–806. doi:10.1126/science.aan4834 28839072 PMC5891123

[26] Turnbaugh PJ, Ridaura VK, Faith JJ, Rey FE, Knight R, Gordon JI. 2009. The effect of diet on the human gut microbiome: a metagenomic analysis in humanized gnotobiotic mice. Sci Transl Med 1:6ra14. doi:10.1126/scitranslmed.3000322 PMC2894525 20368178

[27] David LA, Maurice CF, Carmody RN, Gootenberg DB, Button JE, Wolfe BE, Ling AV, Devlin AS, Varma Y, Fischbach MA, Biddinger SB, Dutton RJ, Turnbaugh PJ. 2014. Diet rapidly and reproducibly alters the human gut microbiome. Nature 505:559–563. doi:10.1038/nature12820 24336217 PMC3957428

[28] Bolnick DI, Snowberg LK, Hirsch PE, Lauber CL, Knight R, Caporaso JG, Svanbäck R. 2014. Individuals' diet diversity influences gut microbial diversity in two freshwater fish (threespine stickleback and Eurasian perch). Ecol Lett 17:979–987. doi:10.1111/ele.12301 24847735 PMC4084827

[29] Baldo L, Riera JL, Salzburger W, Barluenga M. 2019. Phylogeography and ecological niche shape the cichlid fish gut microbiota in Central American and African lakes. Front Microbiol 10:2372. doi:10.3389/fmicb.2019.02372 31681230 PMC6803461

[30] Benson AK, Kelly SA, Legge R, Ma FR, Low SJ, Kim J, Zhang M, Oh PL, Nehrenberg D, Hua KJ, Kachman SD, Moriyama EN, Walter J, Peterson DA, Pomp D. 2010. Individuality in gut microbiota composition is a complex polygenic trait shaped by multiple environmental and host genetic factors. Proc Natl Acad Sci U S A 107:18933–18938. doi:10.1073/pnas.1007028107 20937875 PMC2973891

[31] Bolnick DI, Snowberg LK, Caporaso JG, Lauber C, Knight R, Stutz WE. 2014. Major histocompatibility complex class IIb polymorphism influences gut microbiota composition and diversity. Mol Ecol 23:4831–4845. doi:10.1111/mec.12846 24975397

[32] Bolyen E, Rideout JR, Dillon MR, Bokulich NA, Abnet CC, Al-Ghalith GA, Alexander H, Alm EJ, Arumugam M, Asnicar F, et al.. 2019. Reproducible, interactive, scalable and extensible microbiome data science using QIIME 2. Nat Biotechnol 37:852–857. doi:10.1038/s41587-019-0252-6 31341288 PMC7015180

[33] Douglas GM, Maffei VJ, Zaneveld JR, Yurgel SN, Brown JR, Taylor CM, Huttenhower C, Langille MGI. 2020. PICRUSt2 for prediction of metagenome functions. Nat Biotechnol 38:685–688. doi:10.1038/s41587-020-0548-6 32483366 PMC7365738

[34] Kanehisa M, Goto S, Sato Y, Furumichi M, Tanabe M. 2012. KEGG for integration and interpretation of large-scale molecular data sets. Nucleic Acids Res 40:D109–14. doi:10.1093/nar/gkr988 22080510 PMC3245020

[35] Kumar S, Stecher G, Suleski M, Hedges SB. 2017. Timetree: a resource for timelines, timetrees, and divergence times. Mol Biol Evol 34:1812–1819. doi:10.1093/molbev/msx116 28387841

[36] R_Core_Team. 2021. R: a language and environment for statistical computing:Vienna, Austria. Available from: https://www.R-project.org

[37] Wood SN. 2011. Fast stable restricted maximum likelihood and marginal likelihood estimation of Semiparametric generalized linear models. Journal of the Royal Statistical Society Series B 73:3–36. doi:10.1111/j.1467-9868.2010.00749.x

[38] Shapiro SS, Wilk MB. 1965. An analysis of variance test for normality (complete samples). Biometrika 52:591–611. doi:10.1093/biomet/52.3-4.591

[39] Wilcoxon F. 1945. Individual comparisons by ranking methods. Biometrics Bulletin 1:80. doi:10.2307/3001968 18903631

[40] Watanabe J. 2022. Detecting (non)parallel evolution in multidimensional spaces: angles, correlations and eigenanalysis. Biol Lett 18:20210638. doi:10.1098/rsbl.2021.0638 35168376 PMC8847891

[41] Bolnick DI, Barrett RDH, Oke KB, Rennison DJ, Stuart YE. 2018. (Non)parallel evolution. Annu Rev Ecol Evol Syst 49:303–330. doi:10.1146/annurev-ecolsys-110617-062240

[42] Costello EK, Stagaman K, Dethlefsen L, Bohannan BJM, Relman DA. 2012. The application of ecological theory toward an understanding of the human microbiome. Science 336:1255–1262. doi:10.1126/science.1224203 22674335 PMC4208626

[43] Debray R, Herbert RA, Jaffe AL, Crits-Christoph A, Power ME, Koskella B. 2022. Priority effects in microbiome assembly. Nat Rev Microbiol 20:109–121. doi:10.1038/s41579-021-00604-w 34453137

[44] Ley RE, Peterson DA, Gordon JI. 2006. Ecological and evolutionary forces shaping microbial diversity in the human intestine. Cell 124:837–848. doi:10.1016/j.cell.2006.02.017 16497592

[45] Neu AT, Allen EE, Roy K. 2021. Do host-associated microbes show a contrarian latitudinal diversity gradient? insights from Mytilus californianus, an intertidal foundation host. Journal of Biogeography 48:2839–2852. doi:10.1111/jbi.14243

[46] Baniel A, Amato KR, Beehner JC, Bergman TJ, Mercer A, Perlman RF, Petrullo L, Reitsema L, Sams S, Lu A, Snyder-Mackler N. 2021. Seasonal shifts in the gut microbiome indicate plastic responses to diet in wild geladas. Microbiome 9:26. doi:10.1186/s40168-020-00977-9 33485388 PMC7828014

[47] Videvall E, Strandh M, Engelbrecht A, Cloete S, Cornwallis CK. 2018. Measuring the gut microbiome in birds: comparison of faecal and cloacal sampling. Mol Ecol Resour 18:424–434. doi:10.1111/1755-0998.12744 29205893

[48] Kohl KD, Yahn J. 2016. Eﬀects of environmental temperature on the gut microbial communities of tadpoles. Environ Microbiol 18:1561–1565. doi:10.1111/1462-2920.13255 26940397

[49] Schmidt VT, Smith KF, Melvin DW, Amaral-Zettler LA. 2015. Community assembly of a euryhaline fish microbiome during salinity acclimation. Mol Ecol 24:2537–2550. doi:10.1111/mec.13177 25819646

[50] Sepulveda J, Moeller AH. 2020. The effects of temperature on animal gut microbiomes. Front Microbiol 11:384. doi:10.3389/fmicb.2020.00384 32210948 PMC7076155

[51] Spor A, Koren O, Ley R. 2011. Unravelling the effects of the environment and host genotype on the gut microbiome. Nat Rev Microbiol 9:279–290. doi:10.1038/nrmicro2540 21407244

