# Supplementary material for: The effects of host ecology and phylogeny on gut microbiota (non)parallelism across birds and mammals
Source: mSphere. 2023 Dec 1;8(6):e00442-23. doi: 10.1128/msphere.00442-23 (PMC10732045; doi:10.1128/msphere.00442-23)
Supplement: Supplemental Material — Figures S1-S4 and captions for Tables S1 and S2. [file msphere.00442-23-s0001.docx]

**Supplementary material for:** The effects of host ecology and phylogeny on gut microbiota (non)parallelism across birds and mammals

**Running title:** Gut microbiota (non)parallelism in birds and mammals

**Andreas Härer (AH)^1^, Diana J. Rennison (DJR)^1^**

^1^ School of Biological Sciences, Department of Ecology, Behavior, & Evolution, University of California San Diego, La Jolla, California, USA

**Corresponding author:**

Andreas Härer

School of Biological Sciences, Department of Ecology, Behavior, & Evolution, University of California San Diego

2214 Muir Biology Building

Phone: +1-619-573-8991

E-mail: [ahaerer@ucsd.edu](mailto:ahaerer@ucsd.edu)

# Supplementary Figures


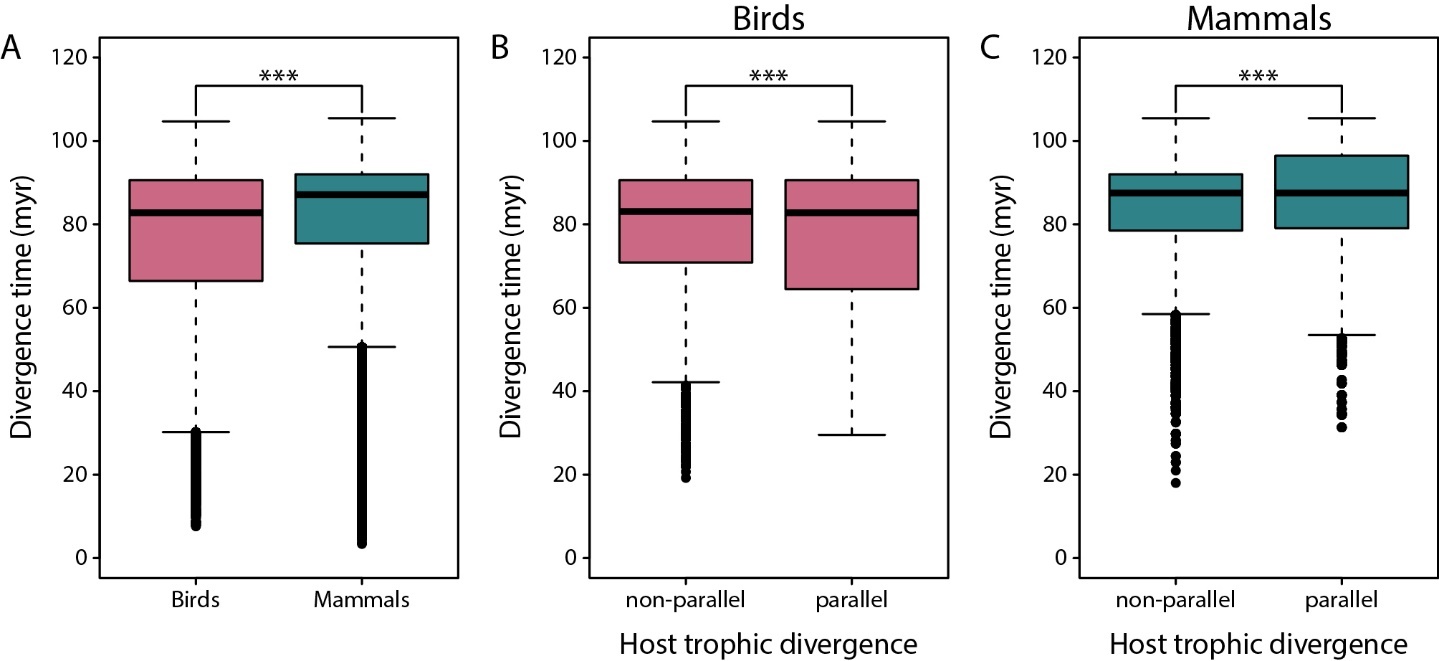


**Figure S1:** Mean divergence times across species pair comparisons differed significantly between birds and mammals (A), but also between the parallel and non-parallel host divergence categories in birds (B) and mammals (C) based on two-sample t-tests. ****P* < 0.001.


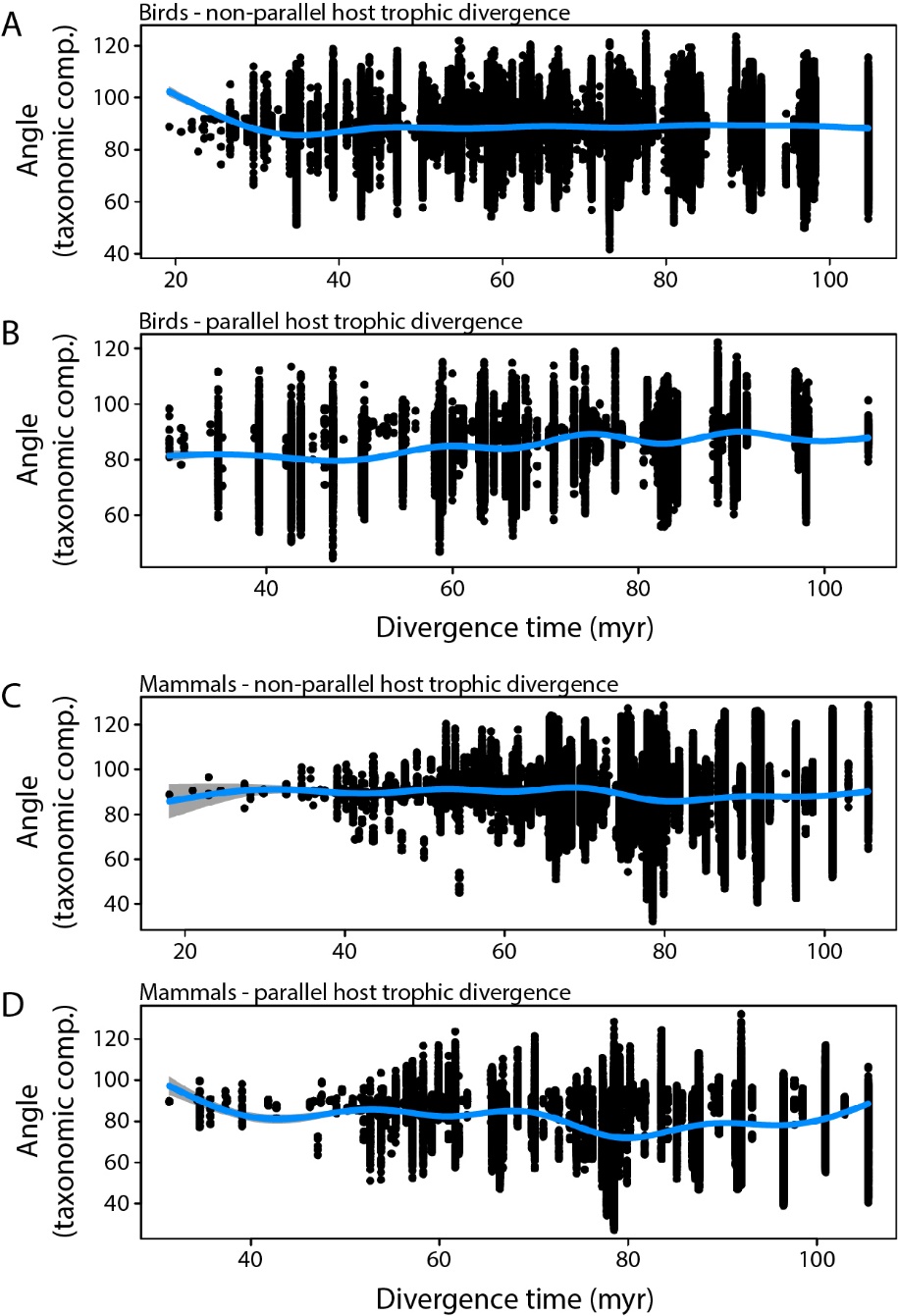


**Figure S2:** When analyzing ‘non-parallel host divergence’ and ‘parallel host divergence’ categories separately, angles based on taxonomic composition were only weakly associated with host divergence time in birds (A&B) and mammals (C&D); generalized additive models are illustrated to identify nonlinear variation. While no general trend was observed, small and large angles were largely missing from comparisons with short divergence times in both birds and mammals.

**
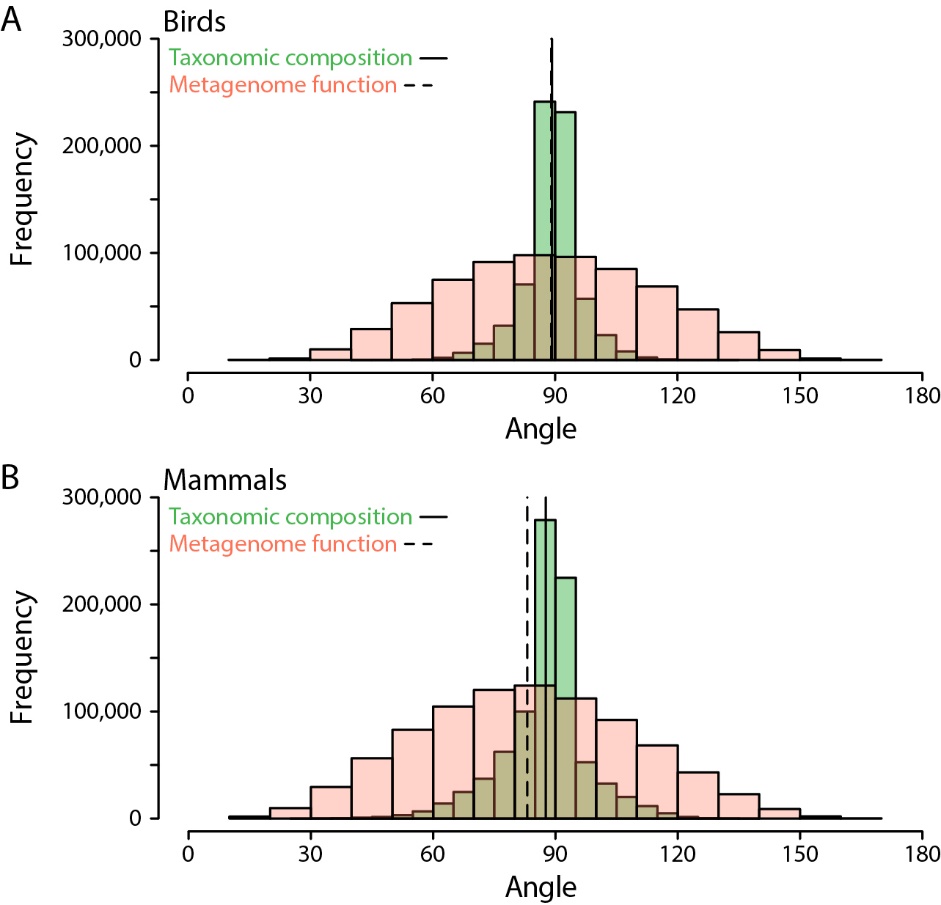
**

**Figure S3:** Frequencies of angles for taxonomic composition and inferred metagenome function across birds (A) and mammals (B) showing a much broader distribution of angles for inferred metagenome function. Mean values are indicated by vertical lines.


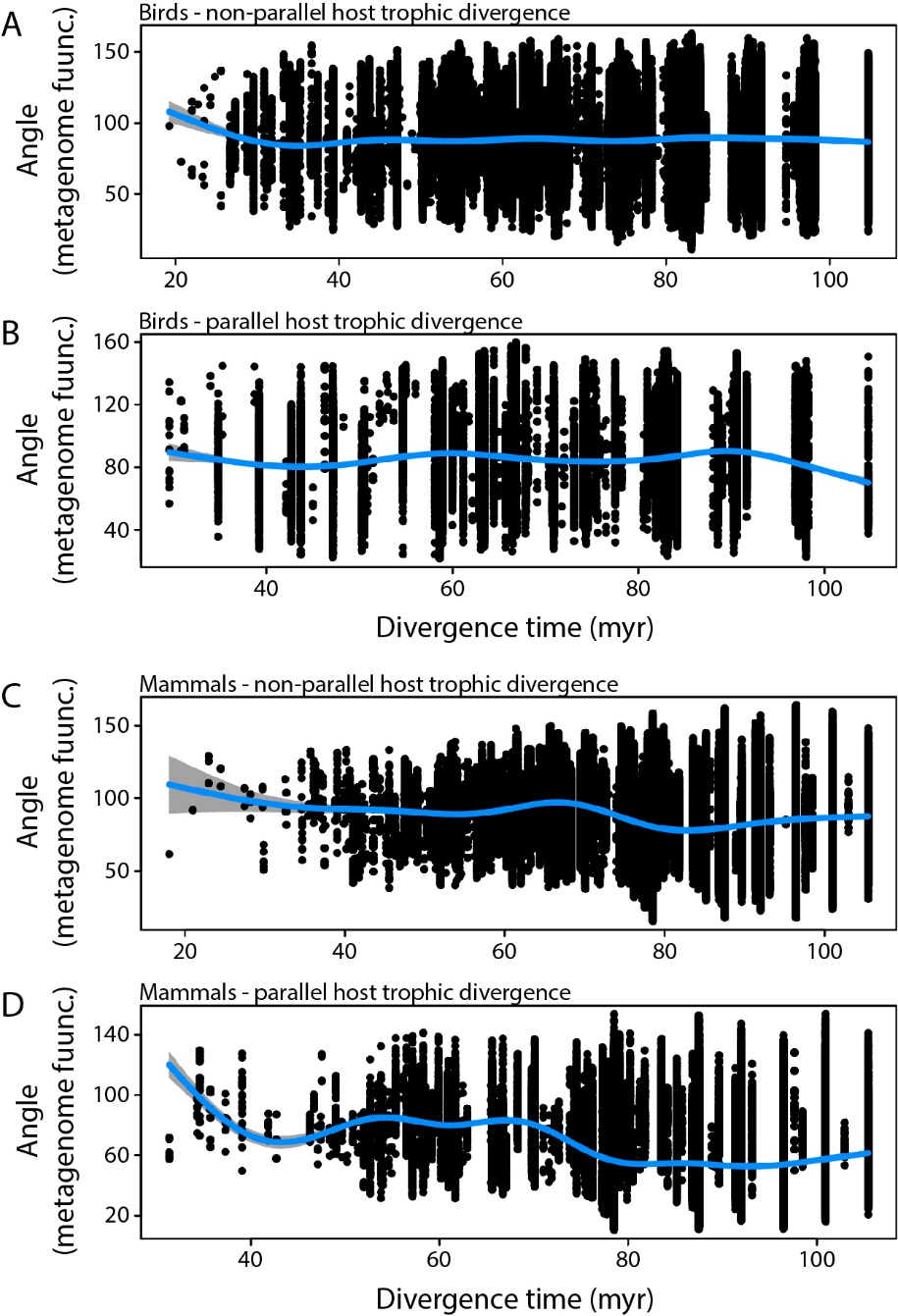


**Figure S4:** When analyzing ‘non-parallel host divergence’ and ‘parallel host divergence’ categories separately, angles based on inferred metagenome function were only weakly associated with host divergence time in birds (A&B) and mammals (C&D); generalized additive models are illustrated to identify nonlinear variation. While no general trend was apparent, small and large angles were largely missing from comparisons with short divergence times in both birds and mammals similar to what we observed for gut microbiota taxonomic composition.

# Captions for Supplementary Tables

**Table S1:** Summary of all avian species analyses in our study including information on sample type, captivity status, flight status as well as the composition of each species’ diet and the diet category which constituted >70% of their diet.

**Table S2:** Summary of all mammalian species analyses in our study including information on sample type, captivity status, flight status as well as the composition of each species’ diet and the diet category which constituted >70% of their diet.
